# Epidemiology, Pathophysiology, and Treatment Strategies of Concussions: A Comprehensive Review

**DOI:** 10.26502/fjhs.178

**Published:** 2024-04-12

**Authors:** Zubair Ahmed, Fihr Chaudhary, Marcel P. Fraix, Devendra K. Agrawal

**Affiliations:** Department of Translational Research, College of Osteopathic Medicine of the Pacific, Western University of Health Sciences, Pomona CA 91766

**Keywords:** Axonal dysfunction, Concussion, Cytoskeletal damage, Electromagnetic field stimulation, Exosomal therapy, Epidemiology, Mitochondrial biogenesis, Neurotrauma, Traumatic brain injury

## Abstract

A concussion is a particular manifestation of a traumatic brain injury, which is the leading cause of mortality and disabilities across the globe. The global prevalence of traumatic brain injury is estimated to be 939 instances per 100,000 individuals, with approximately 5.48 million people per year experiencing severe traumatic brain injury. Epidemiology varies amongst different countries by socioeconomic status with diverse clinical manifestations. Additionally, classifying concussions is an ambiguous process as clinical diagnoses are the only current classification method, and morbidity rates differ by demographic location as well as populations examined. In this article, we critically reviewed the pathophysiology of concussions, classification methods, treatment options available including both pharmacologic and nonpharmacologic intervention methods, etiologies as well as global etiologic differences associated with them, and clinical manifestations along with their associated morbidities. Furthermore, analysis of the current research regarding the incidence of concussion based traumatic brain injuries and future directions are discussed. Investigation on the efficacy of new therapeutic-related interventions such as exosome therapy and electromagnetic field stimulation are warranted to properly manage and treat concussion-induced traumatic brain injury.

## 1. Overview of Traumatic Brain Injury and Concussions

Concussion is a prevalent kind of traumatic injury in the brain. The occurrence of concussions pertains to the rate at which these injuries manifest among a specific demographic. To effectively address concussions, it is important to comprehensively examine multiple aspects of the condition, encompassing the manifestation of symptoms, the trajectory of the damage from its earliest phases to its ultimate stages, and the strategies employed for its management along this progression. In addition, it is imperative to consider the temporal span of concussions, encompassing the period from their initiation to the stage of recuperation, and explore strategies for efficiently managing this duration to mitigate adverse health outcomes. Traumatic brain injury (TBI) is not classified as a disease; yet it is recognized as the leading cause of mortality and disability worldwide among all injuries associated with trauma [1–3]. According to a study conducted by Kamal et al. [4], the World Health Organization (WHO) determined that a significant proportion of fatalities (almost 90%) resulting from injuries are concentrated in low- and middle-income countries. These countries are home to approximately 85% of the world population. This persisting trend is expected to provide a substantial challenge to global health in the foreseeable future. TBI accounts for approximately one-third to one-half of trauma-related deaths and is the primary cause of disability in individuals under the age of forty, with an incidence rate of 15–20 per 100,000 population per year [5]. The economic and social ramifications are significant, since they encompass the direct and indirect expenses associated with acute medical care, rehabilitation, and long-term consequences experienced by affected individuals.

According to Dewan et al. [6], the reported TBIs on a global scale are distributed as follows: mild TBIs account for 81%, moderate TBIs account for 11%, and severe TBIs account for 8%. Numerous instances of cranial trauma, predominantly linked to mild TBI either go unnoticed by healthcare practitioners or remain unreported by affected individuals. Consequently, this phenomenon has been referred to as a “silent epidemic” because of significant gaps in the data provided by epidemiological investigations [7]. In many studies, the overall incidence of severe TBIs is often calculated based on the in-hospital population, while fatal TBIs occurring outside of healthcare facilities are excluded. This exclusion can lead to an incomplete calculation of the frequency of severe TBIs. Therefore, it is possible that TBI is not accurately reflected in data due to its asymptomatic character and the lack of comprehensive injury surveillance or reporting mechanisms in many regions globally [8].

According to the initial findings of the 2010 Global Burden of Disease (GBD) Project conducted by Bryan-Hancock and Harrison [9], the worldwide occurrence rate of TBI was 200 cases per 100,000 individuals annually, resulting in an estimated impact on about 15 million individuals. Dewan et al. [6] quantitatively assessed the prevalence of TBI across various regions defined by the WHO and income classes categorized by the World Bank. These investigators estimated the worldwide prevalence of TBI across all causes and severities to be 939 instances per 100,000 individuals, with approximately 5.48 million people experiencing severe TBI annually, corresponding to a rate of 73 cases per 100,000 individuals. In the United States, a study conducted by Faul et al. [10] estimated that brain injuries had a significant economic impact, amounting to around $75 billion. This estimation takes into consideration several factors such as the expenses associated with treatment, rehabilitation, and the loss of productivity. Additionally, it was found that the average cost per patient was approximately $396,000.

## 2. Epidemiology in High-income countries

In a study for the Italian National reference guideline, the authors presented epidemiological data on TBI generally encountered in high-income nations [11]. The research conducted in this study revealed the presence of two separate peaks in incidence rates. The initial increase was observed during early adulthood, namely between the ages of 16 and 35. This peak was predominantly associated with traffic injuries. The second peak, which exhibited a diminished prominence, was linked to occurrences of falls, and frequently manifested in those aged 70 and above [11]. Majdan et al. [12] undertook extensive research of the epidemiology of TBI in a significant number of European Union member states. The primary objective of their research was to calculate the incidence rates of TBI occurring within hospital settings, as well as the mortality rates associated with TBI across the entire population. Additionally, they aimed to determine the proportion of TBI-related deaths in relation to the overall mortality rates resulting from various types of injuries in European countries. The objective was to generate concise evaluations of these metrics with a specific focus on the European environment. According to Kamal et al. [13], the nations under analysis have established a collective age-adjusted hospital discharge rate of 287 per 100,000 individuals for TBI. Furthermore, it has been documented that these countries have had a pooled age-adjusted death rate of 11.7 per 100,000 individuals because of TBI. TBI constituted around 37% of the overall death rate attributed to various forms of traumas. TBI was shown to be responsible for 42% of the mortality rate in males and 29% in females. A thorough enumeration of 1,375,974 hospital discharges linked to TBI was documented, wherein 61% of these instances pertained to the male gender. Around 55% of the patient cohort was found to be within the age bracket of 0–44 years, whilst roughly 29% of the patients were identified as being 65 years or older. In the study conducted by Kamal et al. [13], it was observed that the proportion of female individuals aged 65 years and above within the latter cohort was higher (41%) in comparison to their male counterparts (21%).

Lawrence et al. [14] did a comprehensive investigation on the epidemiology of TBI across the regions of England and Wales. The present study encompassed a thorough assessment of a nationwide registry that collected data from a prospective, observational investigation centered on individuals admitted to hospitals due to severe trauma. The study has revealed an age distribution that exhibits a unimodal pattern, signifying a prominent concentration of persons within the age range of 80 to 90 years. The demographic in question comprises roughly 20% of the overall population of individuals who have received a diagnosis of TBI. There is a significant increase in the prevalence of severe TBI among individuals aged 20 to 30 years, representing slightly more than 15% of the reported cases. Lawrence et al. [14] present findings from a case series that demonstrate a significant association between age and the etiology of injuries. Individuals in younger age groups exhibit a higher susceptibility to sustaining injuries because of road traffic collisions and incidents of assault. Conversely, with increasing age, there is a concomitant increase in the proportion of individuals who experience injuries resulting from falls of less than 2 meters. Dias et al. [15] did a study examining the occurrence of TBI in adults, utilizing a dataset consisting of 72,865 hospital admissions. The research encompassed a time frame ranging from 2000 to 2010 and centered on the populace of Portugal, which maintained an average population of 10.5 million persons during this duration. Dias et al. [15] reported a sustained decrease in the frequency of admissions related to TBI over a specified duration. Furthermore, it is worth noting that there was a notable increase in the mean age of patients admitted for TBI over the course of the decade, with the average age rising from 52.2 years in 2000 to 65.1 years in 2010. Based on a research study conducted in Finland spanning from 1991 to 2005, a notable increase of 59.4% was observed in the overall count of individuals aged 70 years and beyond who encountered traumatic brain injuries (TBIs). According to a study conducted by Koskinen and Alaranta [16], falls were identified as the predominant external factor contributing to these injuries.

Gardner et al. [17] reported a greater incidence of TBI among the older population. In the year 2013, within the United States, those who were 75 years of age and older accounted for 26.5% of the overall fatalities attributed to TBI, as well as 31.4% of all hospital admissions related to TBI. In a study conducted by Gardner et al. [17], it was determined that the incidence of TBI-related visits to the Emergency Department (ED), hospitalisations, or fatalities in the United States exhibited a higher occurrence among individuals within the age range of 65 to 74 years (greater than 1 in 200) and those aged 75 years or older (greater than 1 in 50) during the year 2013. The research findings also indicated a significantly elevated occurrence of TBI events among individuals in the age group of 0–4 years, with a rate of 1591 per 100,000 individuals. During the period spanning from 2007 to 2013, a significant rise of more than 25% in hospitalizations related to TBI was seen among those aged 75 years and above. The rate of hospital visits associated with TBI among the older population in the United States shown a twofold rise compared to the rate of population growth over the same time frame. Extensive documentation exists regarding the occurrence of TBI among older adults, as evidenced by numerous epidemiological studies undertaken within the United States and across multiple countries with higher-income economies. Numerous studies have continuously documented a notable and increasing prevalence of TBI-related visits to emergency departments, hospital admissions, and mortality rates within the older adult population. Significantly, Haring et al. [18] have substantiated this phenomenon within specific states in the United States and national databases, whilst Iaccarino et al. [19] have documented analogous trends other nations like the United Kingdom, Scotland, the Netherlands, Austria, Canada, and Australia.

## 3. Epidemiology in Low- and middle-income countries

Norton and Kobusingye [20] reported that a significant majority of trauma-related deaths worldwide, specifically 89%, occurred in low- and middle-income nations. According to Saatian et al. [21], there is a higher prevalence of TBI in males compared to females in high-income nations. However, it is important to note that although the male prevalence is frequently documented in high-income nations at a ratio of approximately 2 to 1, this ratio significantly increases to 4 to 1 in low- and middle-income countries such as Tanzania, and even further to 6.5 to 1 in India [22]. In contrast to high-income countries, low- and middle-income countries exhibit a younger population with the highest prevalence of TBI, often ranging between the ages of 28.8 and 33.1, as extensively documented. Reid et al. [23] conducted a retrospective examination of trauma patients who sought medical attention at Kamuzu Central facility in Lilongwe, Malawi, between 2008 and 2015. This facility, which has a capacity of roughly 1000 beds, serves a catchment area with a population of five million individuals. Within the population of trauma patients, it is observed that brain damage constitutes the cause of death for 72% of all individuals who are brought in deceased. According to Reid et al. [23], prehospital mortality was primarily linked to gunshot injuries and incidents involving pedestrians being struck by vehicles.

From March 2013 to December 2016, Saatian et al. [21] conducted a cross-sectional study on patients with head injuries at a prominent provincial university hospital in the northwestern region of Iran. Because of the absence of trauma wards in hospitals located in cities within the province, the responsibility of providing care for trauma patients is delegated to this hospital. Consequently, a total of 9,426 patients diagnosed with TBI were included in the analyses. The present study reveals that the average age of the patients was 29.70±21.46 years, with approximately 73% of the cases falling between the age range of 0 to 40 years. The incidence of TBI in males was found to be twice that of females. According to Saatian et al. [21], a significant proportion of head injuries, specifically 41.75%, were attributed to automobile accidents. Additionally, 30.01% of head injuries were caused by falls of different nature, while attack by bodily force accounted for 7.93% of such injuries. A retrospective study was undertaken by Reza et al. [24] in a prominent trauma center located in the southern region of Tehran.

The study utilized clinical data from a registry comprising 3818 patients who were admitted to the hospital between the years 2009 and 2013. A cohort including 3818 individuals diagnosed with TBI was discovered. The average age of the patients was 38.8±18.7 years. Approximately one third of the patients fell between the age range of 21–30 years, while around one-fifth of the patients were between the ages of 31 and 40 years [24]. The findings of de Almeida et al. [25] indicate a similarity to the present study, as they indicated that individuals aged 20–29 years exhibited the most vulnerability to experiencing a TBI in their research conducted in Brazil. In a report by McCrory et al. [26], individuals with an Injury Severity Score over 9 had a death rate that was directly correlated with the level of economic resources available in their area. Mortality dropped from 63% in low income to 55% in middle-income to 35% in high-income settings. When examining injury severity within the mid-range, it becomes evident that the disparities are much more significant. A study conducted by Iaccarino et al. [19] revealed a six-fold variation in death rates between nations with low income and those with high wealth.

According to the World Health Organization (WHO), a significant proportion of fatalities resulting from injuries, around 90%, transpire in countries categorized as low- and middle-income. These nations are home to approximately 85% of the global population. In low- and middle-income nations, those who typically get TBI are predominantly young adults who engage in activities such as walking, cycling, or riding motorcycles. In geographical areas characterized by elevated levels of armed violence, such as Central America, the Middle East, and Central Africa, TBIs are significantly attributed to incidents involving assault and gunshot wounds. In high-income nations, motor vehicle accidents have emerged as a significant etiological factor for TBI. However, there has been a notable demographic change in the afflicted population, with older age groups now being disproportionately impacted. Falls have been identified as the primary cause of TBI in this context.

## 4. Degree of Concussions

There have been several studies that have linked concussions to a variety of negative health consequences. One area of interest has been the possibility of developing psychiatric diseases in the aftermath of a concussion, namely cognitive impairment. When attempting to evaluate the full impact that concussions have on an individual’s health, the factor is of critical significance and should not be overlooked. When conducting research on concussions, it is necessary to have an in-depth familiarity with the diagnostic processes that are involved. When trying to determine the cause of a patient’s symptoms, doctors will typically combine information gleaned from a clinical exam, various imaging modalities, and neurological examinations. As soon as a diagnosis is obtained, it is of the utmost importance to successfully manage concussions by putting into action a safe and well-organized approach that covers the time beginning with the occurrence of the damage and continuing through the achievement of full recovery. According to research conducted by Baillargeon et al. [27], the brains of young people are more susceptible to sustaining a concussion than the brains of adults, and the time it takes for young people to recover from a concussion may be significantly longer. In addition, the information that is now available suggests that people who have had a concussion are more likely to sustain recurrent concussions, which take place because of a lesser force of impact and call for a lengthier period to recover [28]. According to findings from recent studies [29], females have a greater risk of sustaining a concussion than males do. In addition to this, it has been hypothesized that the vulnerability to sustaining a concussion may be somewhat determined by one’s genetic make-up.

A concussion is a type of brain trauma that affects the entire brain rather than specific regions, and it does not result in any visible neurological abnormalities such as dilation of the pupils or weakening in the limbs. In most cases, the symptoms present themselves in a very subdued manner. These symptoms are marked by sensations of dizziness and the visual sense of light objects, which are sometimes referred to as “seeing stars.” Headaches, dizziness, nausea, and a general sense of unsteadiness were the symptoms that people reported experiencing the most frequently. A small percentage of people report experiencing a loss of consciousness after being in an accident. According to McCrory et al. [26], the initial manifestation of a concussion often presents itself as a mild injury, and full recovery is typically accomplished within a span of time ranging from seven to fourteen days. Due to the lack of a validated biomarker obtained from imaging, blood testing, or computerized neuropsychological screening tools, the only method that can be utilized is clinical diagnosis.

The standard computed tomography (CT) and magnetic resonance imaging (MRI) scans often reveal normal findings following concussions, including cases of recurrent concussions, as stated by McCrory et al. [26]. Ongoing research is being conducted on a wide variety of biomarkers that have been linked to concussions. Some of these biomarkers include the electrophysiology of brain connections or connectivity, as well as irregularities in evoked potentials. Techniques from the field of magnetic resonance imaging (MRI), such as functional MRI, diffusion tensor imaging, and MR spectroscopy, are being utilized in a variety of active research endeavors. An area of research that is now being pursued is the investigation of biomarkers that can be found in serum and cerebrospinal fluid, particularly those that are able to indicate a genetic propensity to concussion. However, as of right now, there is not a single biomarker that has been unequivocally demonstrated as being suitable for this objective [29]. The term “baseline” is being used in the context of the commercial promotion of many computerized neuropsychological tests. However, it is important to remember that these tests, at most, act as supplemental tools to clinical assessment, and that one cannot rely on them to diagnose concussion on their own [30].

## 5. Morbidity Rates of Concussions

Even though a significant amount of research has been conducted on the long-term neurological effects of concussions, we still have a limited grasp of the potential linkages that may exist between concussions and long-term medical and behavioral comorbidities. There is a wide variety of indications and symptoms associated with concussions, including but not limited to disruptions in sleep, cognitive deficits, cephalalgia, vertigo, impulsive conduct, and depressive symptoms. According to McAllister and McCrea [31], there is a growing body of evidence that suggests that a brain injury may have the ability to launch a sequence of degenerative processes that can influence not only the health of the brain but also the health of other physiological systems. The comorbidities that were found in this study included a wide variety of mental conditions, including depressive disorder, anxiety disorder, psychosis, bipolar disorder, sleep problem, and suicidal ideation, intent, and attempt. In addition, substance abuse, which can include drug abuse, opioid abuse, and alcoholism, was recognized as a co-occurring condition in this study. In addition, neurological conditions such as epilepsy and post-traumatic seizures, ischemic stroke, Alzheimer’s disease, and vascular dementia were shown to be present in the patients. In addition, cardiovascular disease (CVD) and the risk factors associated with it were shown to be present as comorbidities. These risk factors include hypertension, hyperlipidemia, obesity, and coronary artery disease. In the end, endocrine problems such as hypothyroidism, hyperthyroidism, pituitary hyperfunction, adrenal hyperfunction, and diabetes mellitus were found, in addition to erectile dysfunction [32].

The long-term health effects of recurrent mild TBI have garnered a lot of attention from the public in recent years. This curiosity has been stoked by a variety of epidemiological and pathological studies that have been carried out on former service members of the armed forces as well as professional American-style football players. According to the findings of these investigations, which were published in 2019 by Grashow et al. [33], persistent multi-system dysfunction and increased mortality rates because of such injuries are present. According to Hammond et al. [34], patients who have had a single moderate or severe TBI have self-reported psychosocial and medical comorbidities. These comorbidities are in addition to the implications of repetitive head injury. The existence of chronic comorbidities was found to have a considerable impact on healthcare expenses, the advancement of recovery, and the chance of mortality in a population of persons who had experienced catastrophic brain damage [34].

In addition, the presence of chronic comorbidities was found to have a significant impact on the progression of recovery. Although the initial neurological symptoms of a concussion often recover on their own within a reasonably short timescale, the long-term impact of concussions on mental, cerebrovascular, and cardiovascular comorbidities remains unknown. This is because concussions are so common. According to Seabury et al. [35], the lack of rigorous long-term monitoring and subsequent evaluation may make the uncertainty surrounding traumatic brain injuries (TBIs) even more pronounced. Izzy et al. [36] demonstrated that individuals who suffered from concussion without pre-existing comorbidities exhibited a heightened susceptibility to the development of a variety of chronic ailments, such as cardiovascular disease (CVD), endocrine disorders, as well as neurological and mental health comorbidities, as compared to control subjects. This increased susceptibility was observed in individuals who had suffered from concussion without pre-existing comorbidities. In addition, the author indicates that the elevated risks of post-concussion were not limited to older age groups; rather, they were also discovered among patients whose ages ranged from 18 to 40 years old. This is an important finding since it shows that the increased risks are not age-related. According to Izzy et al. [36], many of the comorbidities manifested themselves within a median period of less than five years after the occurrence of a concussion. This was shown to be the case for most of the comorbidities.

Following a concussion, it is common for patients to face emotional difficulties, problems sleeping, and behavioral issues, as indicated by research published by Rao et al. [37]. These symptoms are normally of a transient character; nevertheless, there are situations in which they might persist for several months or even for an extended period of time. In a similar vein, it has been discovered that there is a considerable rise in the incidence of reported sleep problems and neuropsychiatric comorbidities, such as anxiety and depression, across various age cohorts in the aftermath of having a concussion [38]. This rise in prevalence has been observed in individuals who have experienced concussions. According to David [39], previous studies have hypothesized the existence of a possible connection between moderate and severe TBI and the development of delayed onset psychosis. On the other hand, no such connection between mild traumatic brain injury and delayed-onset psychosis has been established yet in the research.

Pre-morbid biological risk, family history, and a history of early neurodevelopmental disorders are some of the additional factors that are known to heighten the risk of psychosis and other neuropsychiatric comorbidities. Additional research is required to completely understand the influence of these additional factors, which are known to heighten the risk. According to the findings of previous studies, the investigation of loss of consciousness in populations that are not affiliated with the military has received a relatively small amount of focus. However, the research that has been done to date has shown that a patient’s level of consciousness at the time of injury can serve as a predictive factor for the onset of post-concussion symptoms and depression in the weeks and months that immediately follow a TBI [40]. Following a concussion, Izzy et al. [36] found a significant increase in cardiovascular disease and risk factors for cardiovascular disease, such as hypertension, sleep issues, and obesity, in comparison to the control group. This was notably true for those in the younger age range (18–40 years), who were the focus of the study. People who have had a concussion are more likely to suffer from a variety of negative health effects in the aftermath of the injury, such as an increase in body mass, problems sleeping, persistent tiredness, and a deterioration in their ability to perform daily tasks. According to the findings of Stein et al. [41], individuals who have these features may have a higher risk of acquiring cardiovascular disease and the risk factors that relate to it. There is an elevated risk of cardiovascular disease following a concussion, however the exact cause of this increased risk is not fully understood. It has not been identified whether this increased risk is due to mechanistic reasons, concomitant medical comorbidities, subsequent adjustments in lifestyle that predispose people to greater risk, or other contributing variables. It is possible that all of these could be at play.

Izzy and colleagues [36] found that there was no significant increment in the susceptibility to Alzheimer’s disease and other forms of cognitive impairment within our study population. This observation included people who were above the age of 60. This result may be explained by the fact that the patients in our cohort who were diagnosed with concussion had a healthy baseline condition prior to the onset of the concussion episode that was being studied. Individuals with pre-existing physical and mental health comorbidities, the presence of which is known to increase the risk of Alzheimer’s disease, were not permitted to participate in our study. This is an important point to keep in mind. Izzy and colleagues found that people who had recently suffered concussion had a greater risk of having a stroke. This conclusion is consistent with the findings of more recent research [42], which was also published recently. Even though the mechanisms have not been examined to a great extent, it is possible that this discovery is connected to a worsening of medical comorbidities following injury [39]. In addition, post-concussion mechanisms that have been identified, such as neuroinflammation, vascular dysfunction, and an increased risk of thrombosis, may possibly play a role in the development of the condition [43].

While it is true that some post-concussive symptoms may spontaneously fade without care, it is vital to acknowledge that concussions can lead to substantial and long-lasting implications regardless of the age of the individual who sustains the injury. Izzy et al. [36] have found links between concussions and the chance of developing chronic behavioral health and medical comorbidities, mainly throughout a five-year timeframe after a recorded concussion. These associations were found to be strongest in athletes who had experienced many concussions. When compared to persons who have not suffered concussions, young adults who have experienced head trauma are at an increased risk for developing comorbid conditions.

## 6. Pathophysiology of Concussion

### 6.1 Background

Due to the prevalence of mild traumatic brain injuries (mTBIs), extensive research has been conducted investigating the biochemical and neuro vascular changes associated with it. The combination of studies pertaining to animal mTBI models and a recent increase in clinical trials have strengthened our understanding of the pathophysiology behind mTBIs. Additionally, this deeper insight into biology has led to the development of more effective clinical recommendations for patients. Even with this increased knowledge, specific mechanisms regarding mTBI pathophysiology and their implications in a clinical setting are still readily being explored. Pathways such as excitatory neurotransmitter release, axonal dysfunction, and changes in cerebral vasculature and glucose metabolism occur concurrently during a concussion and are still being examined. The purpose of this review is to critically review the findings regarding mTBI pathophysiology and biomechanics and the instrumental role these principles play among patient populations.

### 6.2 Acute Neurometabolic Cascade

#### 6.2.1 Ionic Flux and Excitatory Neurotransmitter Release

**Figure 1:**
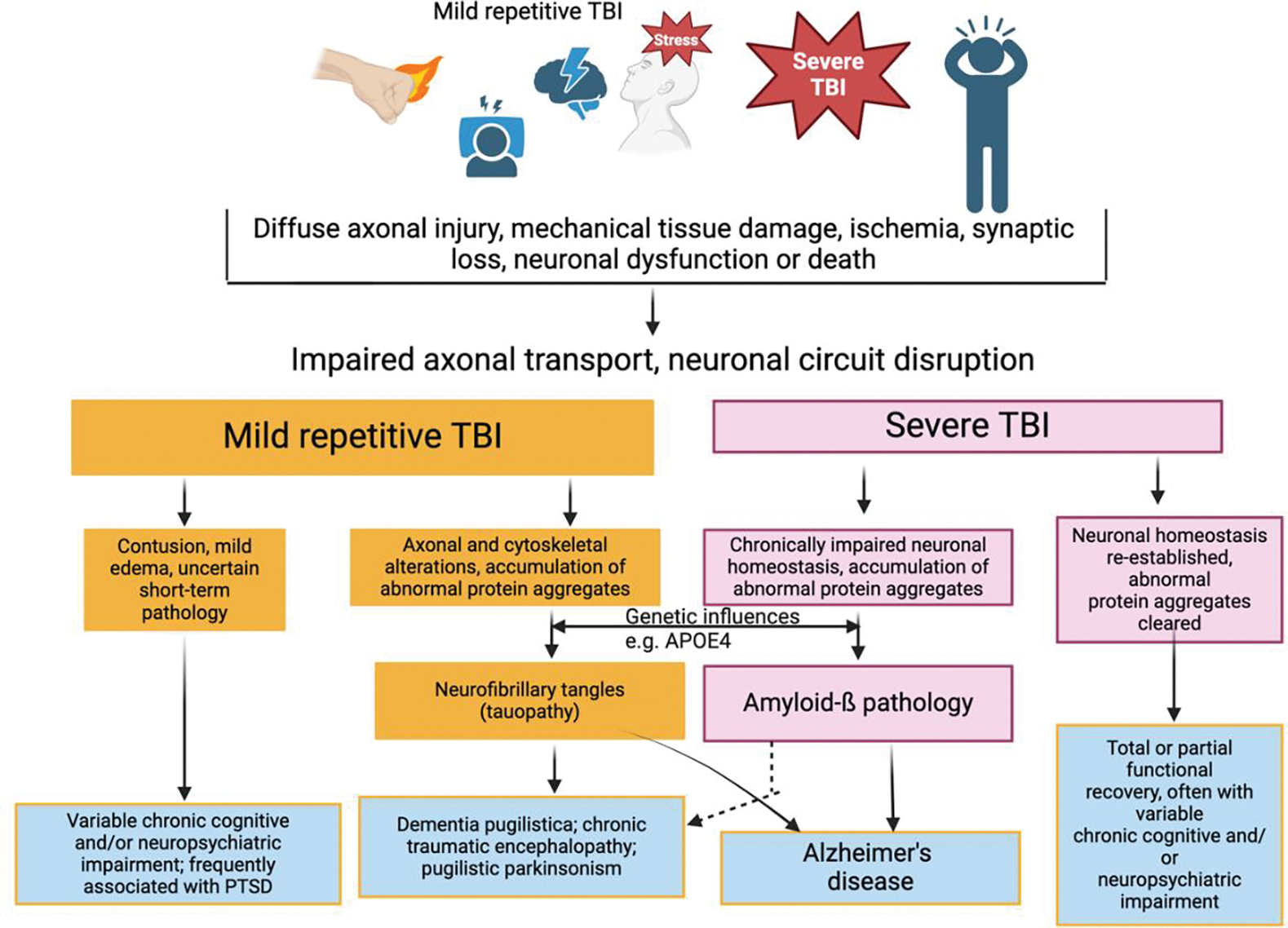
Spectrum of pathological features and outcomes of mild and severe TBI. Abbreviations: APOE, apolipoprotein E; PTSD, post-traumatic stress disorder; TBI, traumatic brain injury.

After a sudden onset of mechanical force, multiple downstream signaling events take place simultaneously that can ultimately lead to a variety of neurological changes such as cognitive impairments, motor deficits, and mental status changes (Figure 1). One notable signaling cascade pathway that is activated after a concussive event is the disruption of cellular ionic homeostasis; thus, initiating a plethora of biochemical alterations such as a sudden neuronal depolarization followed by diffuse neuronal depression [44]. When an mTBI is induced, the plasma membrane undergoes mechanoporation via sheering and stretching forces that cause an influx of sodium and calcium and an efflux of potassium [45, 46]. During depolarization, excitatory neurotransmitters such as glutamate are released. Glutamate triggers the release of potassium through ligand-gated potassium channels and binds to N-methyl-D- aspartate (NMDA) receptors leading to a feedback loop of constant depolarization and hyperexcitability [46]. GABA is an inhibitory neurotransmitter that typically works with glutamate to maintain metabolic activities within the human body. However, due to mTBI, there is an imbalance of these two neurotransmitters that often results in cell damage and neuronal death [47].

Under baseline physiological conditions, glutamate release is highly regulated within the synaptic cleft by astrocytes [48]. However, in response to traumatic brain injuries, it is well established in animal models that there are increased levels of glutamate within the brain due to the stretching and deformation of fibers [49]. In a study conducted in 2012, it was documented that there was a significant increase in extracellular glutamate following diffuse TBI in a rodent model. Using enzyme-based microelectrode arrays (MEAs), they found that glutamate levels were significantly elevated in the striatum specifically, averaging 4.1±0.6 μM compared with sham 2.2±0.4 μM [50]. A recent study from 2017 used proton magnetic resonance spectroscopy(H-MRS) to investigate the concentrations of both glutamate and γ-aminobutyric acid (GABA) at acute and chronic time points post-mTBI in nine athletes compared to nine controls. No differences in glutamate or GABA concentrations were reported in the primary motor cortex. In the dorsolateral prefrontal cortex (DLPFC), glutamate and GABA concentration was lower in the mTBI group compared to controls at 72 hours post-mTBI and GABA levels were also lower 2 weeks post-mTBI. However, the ratio of glutamate to GABA in the DLPFC was higher in the injured group compared to the control at 2 weeks post-injury [51]. These results indicate that the concentrations of both glutamate and GABA post-mTBI are likely region-specific and time-dependent [51]. This is a unique finding because this is one of the first studies to investigate the progression of mTBIs in human populations and the results are different compared to the typical results that are seen in animal models.

**Figure 2:**
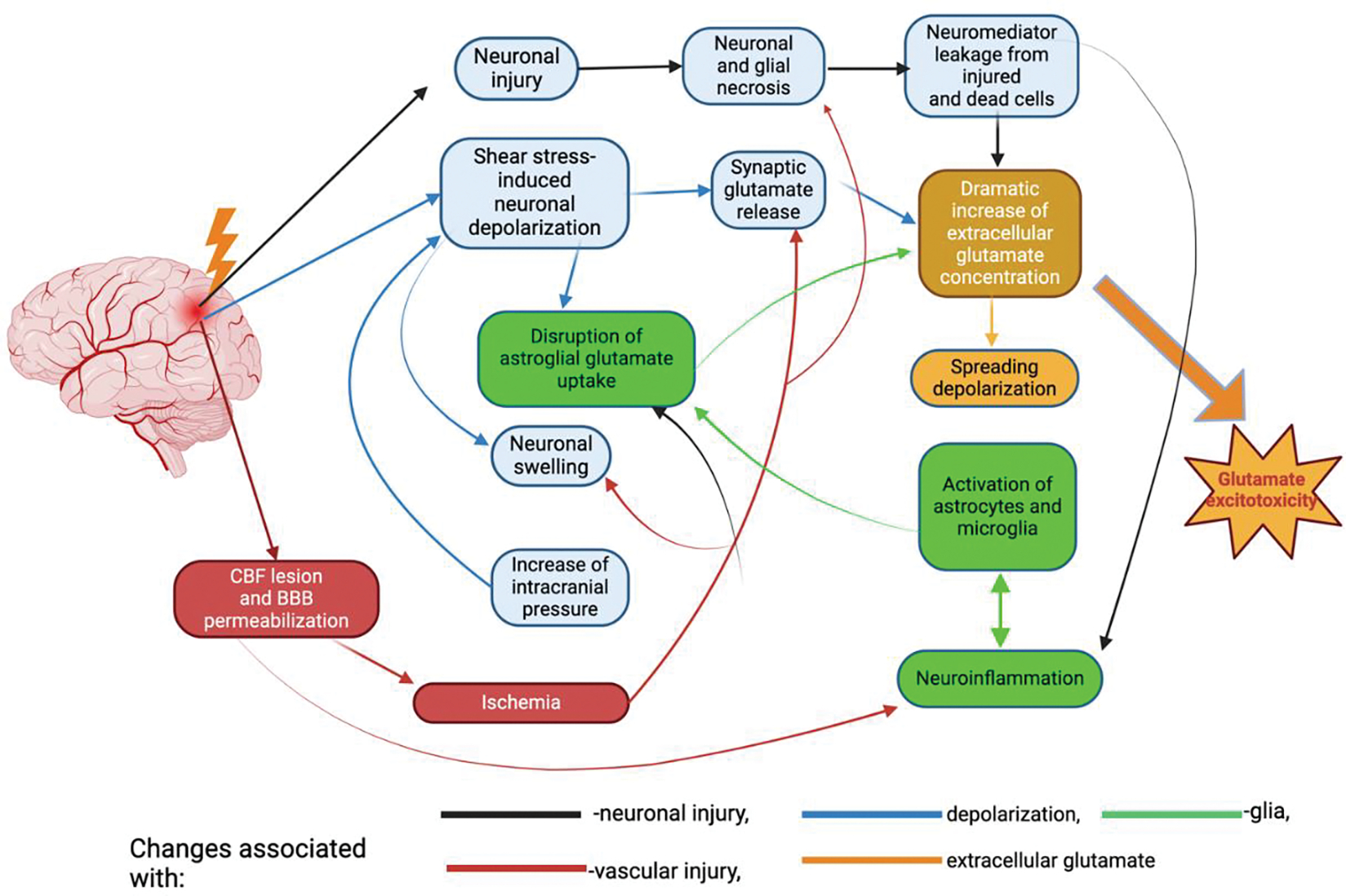
Pathological processes underlying the pathogenesis of traumatic brain injury. The main cause of damage in TBI is the direct mechanical impact, which includes factors such as force, depth of penetration, and extent of damage. This damage occurs within the initial few seconds following the trauma. Secondary damage refers to the longer-term effects that occur over a period of hours to days. These effects are caused by primary damage mechanisms. The factors involved are pathological depolarization resulting from shear stress (shown by blue arrows), alterations in cerebral blood flow dynamics, and neuroinflammatory processes triggered by glial and immune cells entering the brain from the circulation when the blood-brain barrier becomes permeable (indicated by green and red arrows). The delayed neuronal death resulting from TBI is attributed to the excitotoxic effects of glutamate (shown by the yellow arrow).

Due to the elevated levels of glutamate from constant depolarization, NMDA, AMPA, and voltage-gated Na^+^ and Ca^2+^are hyperactive as well, leading to a continuous influx of Na^+^ and Ca^2+^. Under normal physiological conditions, calcium is an important secondary messenger that plays a vital role in maintaining the bioactivity of the cell. However, mTBI causes calcium overload in the cell which is induced by excitotoxicity and leads to eventual cell death [52]. Calcium overload causes cell damage through multiple, different downstream signaling cascades such as free radical production, elevated concentrations of inflammatory mediators, and hyperactivation of cell death signaling pathways [52] (Figure 2).

**Figure 3:**
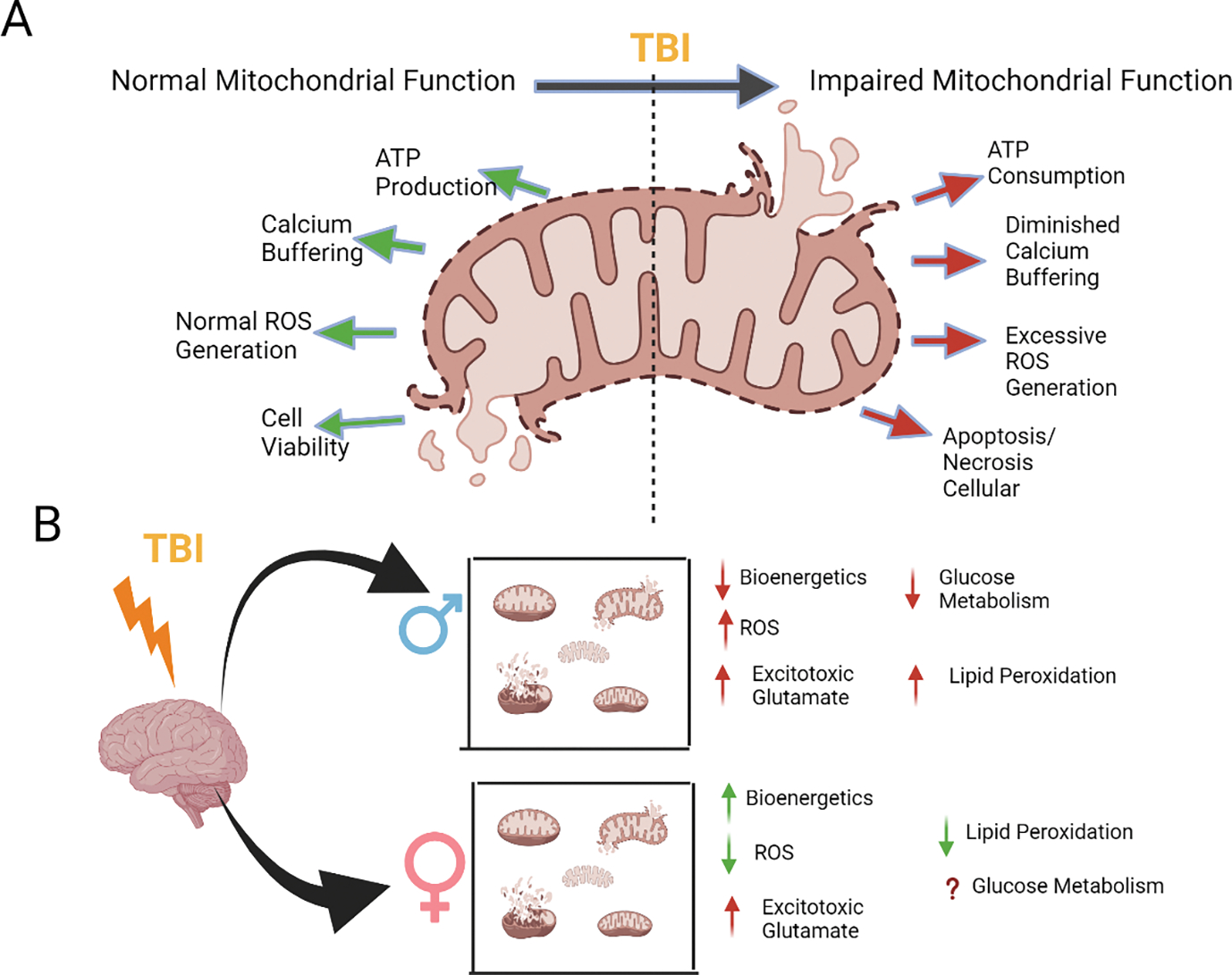
Overview of the mitochondrial dysfunctions caused by traumatic brain injury in comparison to their typical functioning, together with current knowledge regarding sex-specific variations in these dysfunctions. (A) TBI causes a disturbance in the regular functioning of mitochondria. (B) The proven effects of TBI in male models of injury include reduced mitochondrial bioenergetics, increased production of reactive oxygen species (ROS), heightened excitotoxicity and lipid peroxidation, and altered glucose metabolism. Studies focusing on these factors in female models have demonstrated heightened bioenergetics, diminished ROS, elevated acute glutamate levels, and decreased lipid peroxidation. However, the impact of glucose metabolism in females remains uncertain when compared to males.

Initially, the sudden influx of calcium activates calpain and caspases which are cysteine proteases that mediate the degradation of a variety of different metabolic enzymes and membrane proteins [53, 54]. Next, there is an overproduction of reactive oxygen species (ROS) that induces oxidative stress within the cell [55]. Moreover, excessive calcium influx triggers apoptosis through the activation of a variety of pro-apoptotic proteins such as the Bcl-2 family of proteins and leads to ATP depletion [56, 57]. Ultimately, all these cellular pathways that are activated via calcium-induced excitotoxicity led to mitochondrial dysfunction [58]. The mitochondria within the cell become damaged due to excessive calcium uptake through uniporters. This causes decreased amounts of ATP for the cell, apoptosis, or necrosis because of permeabilization of the mitochondrial membrane, and ROS generation [52] (Figure 3).

#### 6.2.2 Energy Crisis

**Figure 4:**
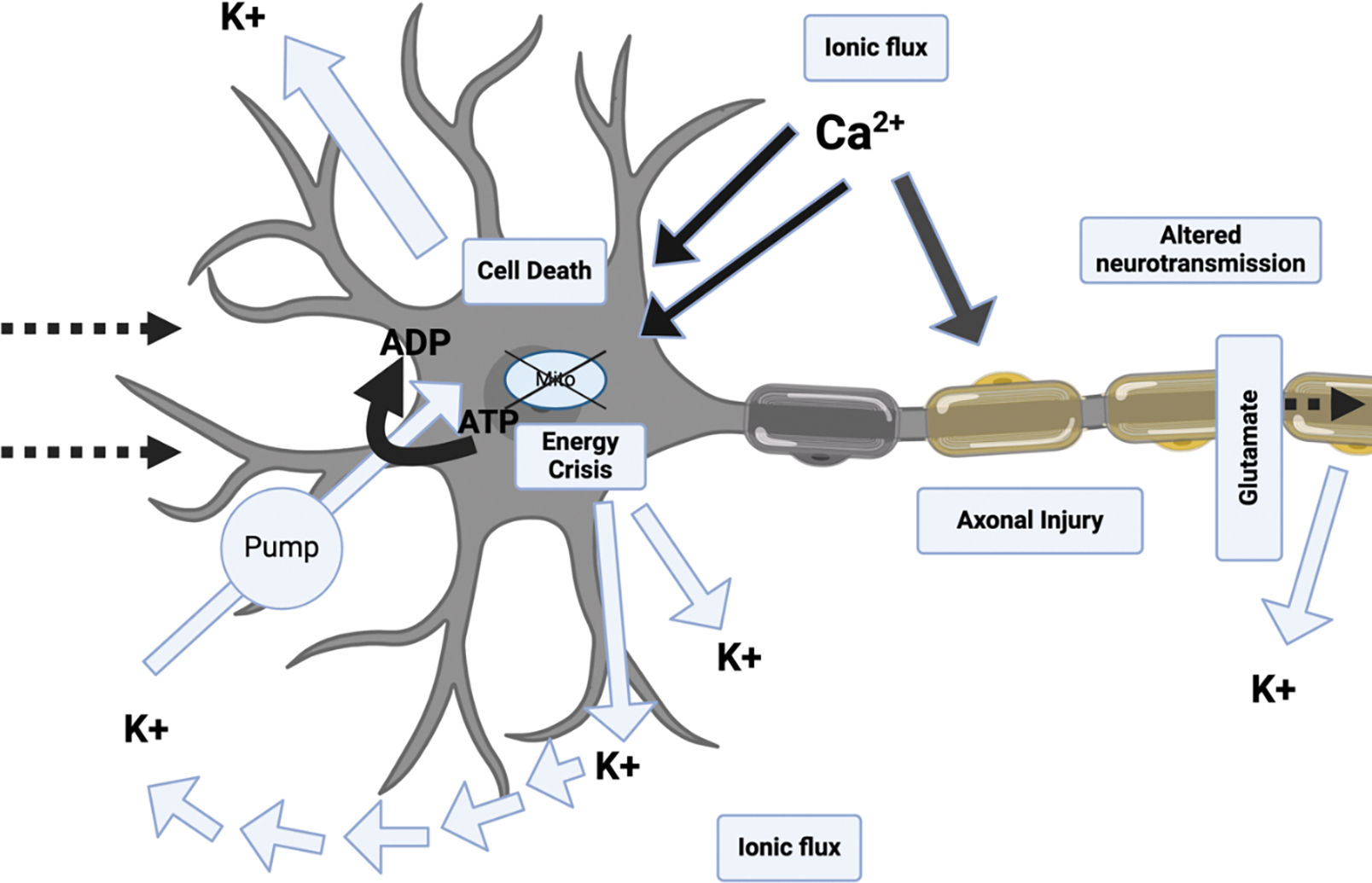
Neurometabolic cascade following traumatic injury. The neurometabolic cascade involves an energy crisis, cytoskeletal damage, axonal dysfunction, altered neurotransmission, inflammation, oxidative stress, and cell death. The brain’s mitochondria lose ATP production, leading to a 7–10-day energy crisis. Cytoskeletal damage affects surrounding structures, causing persistent symptoms. Axonal dysfunction impacts neurotransmission and cognition, causing anxiety, depression, and impaired cognition. Inflammation activates microglia, creating a more inflammatory environment. Oxidative stress, along with altered neurotransmission, energy crisis, and cell death, can lead to long-term damage and symptoms.

Due to the disruptions in ionic and neurotransmitter homeostasis, the cell immediately attempts to restore the balance through membrane ionic pumps. However, these pumps are quickly exhausted because of the excessive energy demands required by the cell [58]. The inability to deliver energy because of altered cerebral blood flow and mitochondrial dysfunction from elevated intracellular calcium, all of which arise from a concussion event, yields an “energy crisis” [59]. These intracellular processes exacerbate the condition of a mTBI-induced and make energy production difficult (Figure 4). In response to an mTBI, animal models have shown increased metabolic energy processes to help achieve homeostasis.

It was reported that there was a 30–46% increase in neuronal glycolytic rate about 30 min after injury which persists for about six hours [60]. Following this glucose hypermetabolism, there is a period of hypometabolism lasting about five to ten 10 days post-injury. Furthermore, due to decreased cerebral blood flow and hence less oxygen being delivered to tissues, anaerobic metabolism is the primary pathway for energy production (Figure 4). Thus, ATP production is reduced, due to ineffective oxidative metabolism, and lactate accumulates leading to an acidic environment [61]. A similar trend was visualized with human participants as well. In a study involving Iraq war veterans who have experienced multiple mTBIs, it was reported that there was a decrease in cerebellar metabolic processes and overall brain hypometabolism after a short period of glucose hypermetabolism [62].

#### 6.2.3 Axonal Dysfunction and Cytoskeletal Damage

The mTBIs that are induced by concussions result in damage to the overall structural integrity of the neuron as well. Specific features of neurons such as axons and microtubules collapsed because of calcium influx [63]. Furthermore, during the trauma itself, a tremendous number of tensile forces are present which impedes cellular transportation and causes swelling of the axon [64]. More specifically, there is an increase in axolemma permeability post-mTBI which is associated with mitochondrial swelling [65, 66]. Furthermore, neurofilament compaction and collapse is a hallmark of mTBI and is typically caused by phosphorylation or by calpain-mediated proteolysis of sidearms [46]. Axon degeneration and microtubule breakdown were shown to occur downstream of intracellular calcium induced by excitotoxicity [67]. Mechanical deformation of the axolemma along with the cytoskeleton components, such as neurofilaments and microtubules, results in the accumulation of beta-amyloid precursor proteins [68]. Moreover, because there are still some intact portions of microtubules, axonal transport still occurs. Thus, there is an accumulation of intracellular organelles at the site of damage resulting in focal swelling and leading to secondary axotomy [46, 69] (Figure 4).

## 7. Clinical Translation of Concussion and Evaluation

The evaluation of concussion must consider numerous symptoms given the mechanism of injury and pathophysiology. These are discussed below. Regardless of the presenting symptoms, patients should be assessed by a licensed health care professional. This can be at the sideline of an athletic event or in the emergency department or a physician’s office. The cornerstone of evaluation relies on mental status and neurological testing, which incorporates the Glasgow Coma Scale (GCS) in an acute setting and the evaluation of cranial nerve function, motor strength, balance, and coordination. Evaluation should also include a standardized inventory when appropriate, such as the Standardized Assessment of Concussion (SAC), which can be particularly helpful for the sideline evaluation of athletes. The Sport Concussion Assessment Tool (SCAT) is particularly useful in the acute phase of injury, especially within the 72 hours of injury [70]. Additional testing includes computed tomography (CT) and magnetic resonance imaging (MRI). CT without contrast is recommended in patients with acute concussion or mild traumatic brain injury, especially in those patients with a GCS score < 15, neurological deficits, signs of skull fracture or amnesia. MRI is typically not used in the acute setting and is recommended for patients who demonstrate atypical recovery following concussion or if there are abnormal neurological deficits with a normal CT scan [71].

### 7.1 Headaches

According to the International Classification of Headache Disorders (ICHD-3), post-traumatic headache (PTH) is defined as a type of secondary headache that typically occurs seven days after trauma or injury, or within seven days after recovering consciousness [72]. PTH is one of the most common complaints after an mTBI with a prevalence of 30–90% [72]. Some common symptoms of PTH are nausea, vomiting, sensitivity to light, and fatigue [73]. Even though PTH is described as a secondary headache, the symptoms and clinical presentation share many similarities with a primary headache. For example, PTH can be classified into migraine-like or tension-type headaches [74]. Additionally, PTH can be categorized as acute if the symptoms resolve within three months or chronic if it lasts longer than that. PTH accounts for 4% of all secondary headache disorders [75]. In a study from 2014, the prevalence of headaches was investigated immediately, three months, six months, and 12 months following mTBI. Out of the 212 participants in the study, all of whom were enrolled in one-week post-injury, 54% of individuals reported having new or worsening immediately after mTBI, 62% at three months, 69% at six months, and 58% at 12 months [76]. When a PTH becomes chronic, it can result from either peripheral or central origin. As it pertains to peripheral origin, chronic PTH can be caused by chronic stimulation of nociceptors in peripheral tissue and/or damage to cranial nerves [77]. In central origin, chronic PTH can result from impairments in the spinothalamic and thalamocortical pathways [77].

### 7.2 Cognitive Deficits

It is well-known that concussions in general can have detrimental effects on cognition. However, the cognitive deficits regarding sport-related concussions (SRC) specifically have been more widely studied due to its importance in the sports medicine community and the impact it has on athletes. Furthermore, due to the variety of possible symptoms, concussion detection and diagnosis is very difficult. Additionally, trusting a concussed athlete regarding their symptoms is not always a reliable metric; thus, a multi-faceted approach using neurocognitive testing, such as paper-and-pencil neuropsychological tests and computerized cognitive testing, and electroencephalography (EEG), are utilized to assess the management of concussion and the degree of severity of symptoms [78, 79]. In a recent study, the acute effects and recovery following SRC were observed [80]. Out of 396 high school and college football players, 28 athletes with concussions were chosen and 28 were controls. All participants underwent baseline testing on measures regarding cognitive function using quantitative electroencephalography (QEEG). Neurocognitive testing and QEEG were used on the day of injury, eight days after injury, and 45 days after injury. Upon analysis of the results, the injured population experienced more significant concussive symptoms during the first three days after injury, but the symptoms resolved by day five and day eight. Significant abnormalities in electrical brain activity were also visualized amongst the injured population on the day of injury and eight days following concussion, but not 45 days post-injury. Furthermore, the injured group performed worse on neurocognitive testing compared to controls on the day of injury, but no significant differences were found eight- or 45-days post-injury [80]. Based on QEEG results, it was concluded that the physiological recovery after a concussion may be longer than clinical recovery which was assessed using neurocognitive testing.

In addition to the acute effects of concussion, long-term effects have been studied as well. It was reported that retired athletes with SRC had significant cognitive deficits in verbal memory, delayed recall, and attention compared to a non-injured group [81]. Moreover, contact sports athletes, such as players from the National Football League and the National Hockey League, performed worse on Letter Fluency testing and immediate recall compared to non-contact athletes which further highlights the long-term cognitive deficits after a concussion [82]. Furthermore, athletes involved in high-intensity activity following a concussion performed worse on neurocognitive testing while those involved in moderate-intensity activity performed the best [83].

### 7.3 Motor Impairments and Slowed Reaction Times

Motor deficits are also commonly seen following concussions. In a recent study from 2019, adolescent athletes diagnosed with an SRC completed a single-task and dual-task standing and walking within 14 days of the concussion and were compared to non-concussion individuals. Gait and quiet stance root mean square (RMS), which is a measurement of balance, were metrics used to evaluate motor deficits. The study found that the injured population walked slower than the control, but there were no differences in quiet stance RMS between the two groups [84]. Thus, this study indicates that certain tasks that require greater motor coordination may demonstrate greater deficits after a concussion. Reaction time (RT) is another metric that is utilized to assess the severity of a concussion. One study measured the effects of concussion on reaction time by using a clinical test that involved grabbing a falling measuring stick [85]. A group of concussed athletes and non-concussed(control) participants completed this test at baseline and within 48 hours post-injury. Upon analysis of the results, there was a significant difference in the reaction times between both groups. In the concussed group, there was a significant prolongation from baseline to post-injury. Improvement was noted in the non-concussed group between the two intervals [85]. Another study found that the reaction time, after a concussion compared with baseline, was 26 milliseconds(ms) slower at 48 to 72 hours post-injury, 18 ms slower seven days after concussion, and 9 ms slower ten days post-injury [86]. Ultimately, reaction time did not return to baseline until 14 days following a concussion.

## 8. Management of Concussion Symptoms

In most nations, it has been customary for individuals who have had a concussion to be promptly withdrawn from any ongoing involvement and undergo an assessment conducted by a qualified medical practitioner. The initial endorsement of this stance can be traced back to the Canadian Academy of Sport Medicine in 2000, as documented in the publication titled “Guidelines for Assessment and Management of Sport-Related Concussion” [87]. Subsequently, this viewpoint has been consistently supported by esteemed professionals at many worldwide consensus conferences dedicated to the topic of concussion. The primary components of initial management involve the use of measures to address removal from play, job or school, and rest. According to the collective agreement among specialists, the concept of “rest” has been expanded to encompass not only physical but also cognitive activity [26]. The incorporation of mental rest signifies a significant shift in the realm of administration. Early management has not demonstrated any viable treatment options other than rest. Removal from play until symptoms resolve maybe a consideration in the prevention of second impact syndrome. Although this is considered a rare and not well understood condition, it potentially exposes a patient or athlete to more serious neurological sequelae when they are still symptomatic from a prior concussion or mild traumatic brain injury [88].

In cases when post-concussion syndrome is present, characterized by the prolonged persistence of concussion symptoms for a duration of weeks or months, it is advisable for the athlete to refrain from resuming participation in sporting activities. When post-concussion syndrome persists for an extended duration of many months, it becomes necessary to contemplate the permanent exclusion of individuals from participating in collision sports. It is advisable to consider such a course of action in cases with enduring cognitive impairment. In such instances, it is imperative to conduct comprehensive and rigorous neuropsychological assessments. According to Cantu and Register-Mihalik [89], the permanent exclusion from collision sports is determined by several factors, including the presence of enduring neurological impairments, movement abnormalities, and the observation of lesions on CT or MRI scans.

Physicians can further primary prevention efforts by campaigning for the implementation of more comprehensive legislation, like the existing measures in place across several states in the United States. These legislative measures aim to identify and prevent brain injuries that occur during sports and recreational activities. One potential measure to consider is the implementation of compulsory educational programmes focused on concussions and optimal management strategies, specifically tailored for children and adolescents participating in team collision sports. These programmes would aim to educate not only the young athletes themselves, but also their parents, coaches, trainers, and referees. Helmets have been shown to effectively mitigate severe brain injuries, such as cerebral lacerations and intracranial hematomas. However, it is important to note that existing helmet designs do not provide complete protection against all concussions, as they are unable to fully reduce rotational acceleration of the brain [90].

## 9. Treatment

### 9.1 Current Treatment Options

The continuous rise of concussions every year coupled with the fact that there are still few evidence-based treatment options is a prominent health concern in the medical community. As a result, it is difficult to provide effective symptom management recommendations to patients who are suffering from post-concussion syndrome (PCS). For reference, the World Health Organization defines PCS as a syndrome that occurs following head trauma and includes several disparate symptoms such as headache, dizziness, fatigue, irritability, difficulty in concentration and performing mental tasks, impairment of memory, insomnia, and reduced tolerance to stress, emotional excitement, or alcohol [91]. Pharmacological and non-pharmacological therapies have been implemented to treat PCS such as prescribed rest and analgesics.

#### 9.1.1 Prescribed Rest

Prescribed rest is one of the most common clinical recommendations for concussions. One study evaluated the changes in brain activity in concussed male athletes compared to individuals who had no history of concussions. Through the utilization of multiple verbal and visual working memory tasks, levels of brain activity were examined with functional magnetic resonance imaging (fMRI). The study found that levels of activation in the concussion group were significantly higher than in the non-concussion group [92]. These elevated levels of activity are an indicator of the brain’s compensatory mechanism following a loss of function that is seen after a concussion. This excessive activation leads to the overproduction of ROS and results in more oxidative stress. Furthermore, due to the disruption in chemical homeostasis caused by an mTBI, a concussed brain is more susceptible to injury [93]. Thus, these results suggest that rest is required to combat the stress that is placed on the body following mTBI.

Although rest is important for recovery, studies have shown that it is only beneficial in the acute phase, and it can delay recovery in the long-term. The consensus statement from the 5th International Concussion Conference held in Berlin in 2016 was that cognitive and physical rest should last 24–48 hours post-injury followed by a gradual increase in recovery [94]. This ramp-up in activity is a part of the current guidelines to promote optimal recovery of symptoms and help the patient return to pre-injury activity levels. One study analyzed the effects of long-term rest on individuals’ post-concussion. Children of ages 11–22 and present in the Emergency Room 24 hours after a concussion participated in this study. It was reported that individuals who were strictly resting for more than 10 days after injury had worse symptoms and took longer to recover [95]. Another study also found that prescribed rest for more than 10 days without any activity led to more symptoms and slower recovery [96]. Furthermore, it was shown that individuals benefited from prescribed cognitive and physical rest if it was less than one week [97, 98]. Moreover, moderate levels of activity over the course of a month following injury had better neuropsychological outcomes than those with little activity [99]. Ultimately, it can be concluded rest is beneficial in the short term, but there should be an increase in physical activity in the long term for optimal recovery post-concussion [100].

#### 9.1.2 Analgesics

As discussed above, post-traumatic headache (PTH) is one of the most common complaints after a concussion. Typically, analgesics are prescribed by healthcare providers to help alleviate the symptoms. However, excessive use of these medications can cause a medication overuse headache (MOH) [101]. One study investigated whether analgesic overuse contributes to PTH. Out of 54 concussion patients who met the criteria for MOH, 68.5% had complete resolution of their PTH after stopping analgesic treatment. Thus, it was concluded that overuse of analgesics post-concussion may contribute to PTH [101].

### 9.2 Future Treatment Strategies

#### 9.2.1 Exosome Therapy

**Figure 5:**
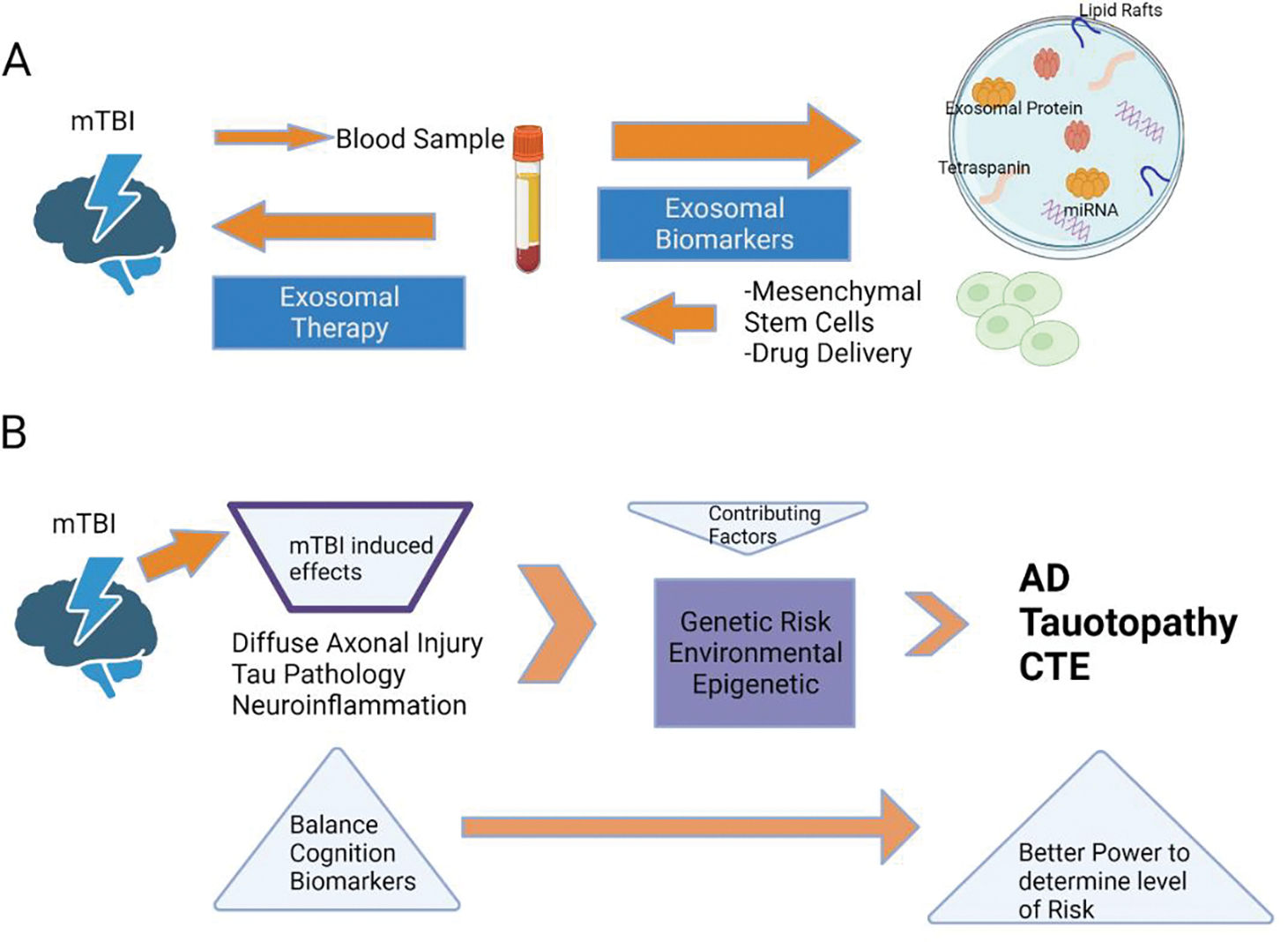
A schematic depicting the potential biological and medicinal applications of exosomes (A) and a suggested procedure for identifying the genetic, epigenetic, and environmental components that contribute to the consequences experienced by humans following a concussion (B). (A) In order to identify brain-related components, such as proteins and miRNAs, that are involved in either short-term or long-term post-concussion brain modifications, it is possible to purify either ADEs or NDEs from blood samples. Exosomes derived from enriched stem cells, including mesenchymal stem cells, can be transported via the bloodstream to target regions of the brain impacted by TBI, according to new research in the cancer field (B). Multiple harmful pathogenic consequences are induced in the brain by mild traumatic brain injury (mTBI). These impacts, in addition to those caused by genes, the environment, and epigenetics, increase the likelihood of neurodegeneration in old age. Chronic traumatic encephalopathy (CTE), Alzheimer’s disease (AD), tauopathy, and other neurodegenerative disorders may manifest years after mild traumatic brain injury (mTBI) has occurred. The prognosis following mTBIs might be impacted by genes, epigenetics, and the surrounding environment. A more thorough evaluation including improved cognitive and balance tests in addition to biomarker studies after mTBI may, however, be able to identify those people at heightened risk of neurodegeneration because of mTBI.

Exosomes are nano-sized extracellular vesicles that contain proteins, lipids, and nucleic acids and play an important role in mediating intracellular communication. They are typically generated in response to TBIs and have both protective and pathological effects that lead to further development and progression of the injury [102]. Depending on the context, exosomes can promote angiogenesis, stromal remodeling, signal pathway activation through growth factors, chemoresistance, and immunologic activation and mediate inflammatory responses [102]. Given the significant roles of exosomes, they have been shown to be used as diagnostic biomarkers for TBI as well (Figure 5).

Due to the tremendous potential of exosomes in the process of alleviating the pathophysiological effects of TBI, some studies have begun investigating the use of exosomes as a possible treatment. Thus far, mesenchymal stem cell (MSC)-derived exosomes have demonstrated therapeutic effects after experimental brain injury [103, 104]. One study reported that intravenous delivery of MSC-derived exosomes exhibited improved functional recovery and promoted neuroplasticity in a rodent model following TBI (50). Another study examined the effects of intracerebroventricular microinjection of human adipose MSC-derived exosomes in a weight-drop-induced TBI rodent model. In addition to promoting functional recovery, MSC-derived exosomes suppressed neuroinflammation, decreased neuronal apoptosis, and increased neurogenesis [104]. Based on these results, MSC-derived exosomes have exhibited promising results as a potential therapy for TBI (Figure 5), but some limitations remain and further carefully planned studies are needed. The mechanism of action of exosomes regarding how they improve recovery and cross the blood-brain barrier remains unclear. Additionally, identifying the optimal source of cells used for creating exosomes and the intracellular contents is important. Furthermore, establishing an appropriate dose, time window to administer exosomes post-injury, and optimal route of administration are all essential factors to consider when developing an effective treatment. Thus far, exosome therapy has primarily been studied in animal models. However, by taking these next steps and answering such questions, exosomes can soon be studied in clinical populations.

#### 9.2.2 Electromagnetic Field Stimulation to Heal Concussion Injury

The use of electromagnetic fields (EMF) is a novel and progressive area of medical research in various conditions, including ischemic strokes, spinal cord stimulation to relieve pain, bone stimulation to heal non-unions, tremors to calm the hands, and in the heart to capture a beat [105–108]. However, the effects of EMF on the healing of concussion or other traumatic brain injuries are unknown.

EMF stimulation may modulate inflammatory response by inhibiting cellular infiltration, controlling the activity of astrocytes and microglia, and reducing calcium influx, reactive oxygen radicals, and inflammatory cytokines to enhance mitochondrial biogenesis, induce remyelination and thus improve neuronal recovery [3, 109]. In our recent pilot studies, we for the first time, effectively measured neuronal EMFs with an engineered Mu-metal and copper mesh-shielded helmet in a swine model of TBI [110–114]. We identified the measurement of cortical function in swine using novel induction sensors and shielding isolated to a helmet and EMF channels, and patterns within EMF were identified pre- and post-injury for altered neural circuits that were measured using the sensors continuously, non-invasively, and in real-time [110–113]. Indeed, EMF stimulation resulted in the early and durable recovery of neuronal circuit-driven EMF patterns [112, 113]. Histological studies supported the preservation of neuronal tissue morphology with the reduction of inflammatory markers at the transcriptional and translational levels [114, 115]. We also identified differentially expressed genes (DEGs) that were significantly upregulated in the injured brain tissue and the EMF stimulation showed an effect on their expression profile [9,10]. Transcriptomic analysis revealed several DEGs including INSC, TTR, CFAP126, SEMA3F, CALB1, CDH19, and SERPINE1 [116]. These genes are associated with immune cell infiltration, myelination, reactive oxygen species regulation, thyroid hormone transportation, cell proliferation, and cell migration. There was a time-dependent effect of EMF stimulation on the gene and protein expression. The findings support the beneficial effect of EMF stimulation in the repair process following TBI either due to concussion or other trauma. However, additional well-controlled pre-clinical and clinical studies are warranted.

## 10. Conclusion

Concussions and other traumatic brain injuries is a leading cause of mortality and disability worldwide, with 90% of fatalities concentrated in low- and middle-income countries. TBI accounts for one-third to one-half of trauma-related deaths and is the primary cause of disability in individuals under forty. In high-income countries, incidence rates are higher, with two peaks observed during early adulthood and falls. Concussions can lead to cognitive impairment and longer recovery times, with younger individuals being more susceptible and females having a higher risk of sustaining one than males. Understanding the diagnosis and management of symptoms is crucial for effective concussion management. Research on the long-term neurological effects of concussions is limited, but evidence suggests brain injuries can lead to degenerative processes affecting other physiological systems and can have detrimental pathophysiological effects as well. Disruptions to ionic homeostasis and axonal deformation caused by a concussion can lead to an energy crisis within the body. As it pertains to clinical manifestations of concussion, headaches are the most common complaint post-injury. Cognitive deficits as well as decreased reaction times are also visualized post-injury which typically resolve over time. The most common current treatment recommendations include prescribed rest and analgesics. Although prescribed rest is beneficial at first, studies have demonstrated that a ramp-up in activity 48 hours post-injury can help accelerate recovery. Additionally, analgesics have been shown to be a main contributor to the development of headaches as well. Exosome therapy has thus far indicated promising results, but further research is needed to analyze the efficacy in clinical populations. The preliminary studies in swine model of traumatic brain injury strongly support the beneficial effect of EMF stimulation in the healing of injury due to concussion and this could prove to be a revolutionary treatment in managing patients following traumatic brain injury due to concussion or other trauma.
